# Supplementary material for: Victory above all: the weight loss practices and perceptions of Chinese male kickboxers
Source: PeerJ. 2025 Jul 9;13:e19709. doi: 10.7717/peerj.19709 (PMC12255242; doi:10.7717/peerj.19709)
Supplement: Supplemental Information 1 [file peerj-13-19709-s001.pdf]

STROBE Statement—checklist of items that should be included in reports of observational studies

	Item No.	Recommendation	Page No.	Relevant text from manuscript
**Title and abstract**	1	(*a*) Indicate the study's design with a commonly used term in the title or the abstract	1	Victory above all: the weight loss practices and perceptions of Chinese kickboxers
		(*b*) Provide in the abstract an informative and balanced summary of what was done and what was found	2	Line 19-49
**Introduction**				
Background/rationale	2	Explain the scientific background and rationale for the investigation being reported	4-6	Line 52-118
Objectives	3	State specific objectives, including any prespecified hypotheses	7	Line 118-121
**Methods**				
Study design	4	Present key elements of study design early in the paper	7	Line 125-130
Setting	5	Describe the setting, locations, and relevant dates, including periods of recruitment, exposure, follow-up, and data collection	8-9	Line 133-152 and Line 176
Participants	6	(*a*) *Cohort study*—Give the eligibility criteria, and the sources and methods of selection of participants. Describe methods of follow-up<br>*Case-control study*—Give the eligibility criteria, and the sources and methods of case ascertainment and control selection. Give the rationale for the choice of cases and controls<br>*Cross-sectional study*—Give the eligibility criteria, and the sources and methods of selection of participants	8	Line 144-147
		(*b*) *Cohort study*—For matched studies, give matching criteria and number of exposed and unexposed<br>*Case-control study*—For matched studies, give matching criteria and the number of controls per case		
Variables	7	Clearly define all outcomes, exposures, predictors, potential confounders, and effect modifiers. Give diagnostic criteria, if applicable	Not applicable	Not applicable
Data sources/ measurement	8*	For each variable of interest, give sources of data and details of methods of assessment (measurement). Describe comparability of assessment methods if there is more than one group	8-9	Line 157-176

Bias	9	Describe any efforts to address potential sources of bias	9	Line 170-176
Study size	10	Explain how the study size was arrived at	7	Line 125-127
Quantitative variables	11	Explain how quantitative variables were handled in the analyses. If applicable, describe which groupings were chosen and why	10	Line 183-188
Statistical methods	12	(*a*) Describe all statistical methods, including those used to control for confounding	9-11	Line 179-211
		(*b*) Describe any methods used to examine subgroups and interactions	9-11	Line 179-211
		(*c*) Explain how missing data were addressed	Not applicable	Not applicable
		(*d*) *Cohort study*—If applicable, explain how loss to follow-up was addressed<br>*Case-control study*—If applicable, explain how matching of cases and controls was addressed<br>*Cross-sectional study*—If applicable, describe analytical methods taking account of sampling strategy	Not applicable	Not applicable
		(*e*) Describe any sensitivity analyses	Not applicable	Not applicable
**Results**				
Participants	13*	(a) Report numbers of individuals at each stage of study—eg numbers potentially eligible, examined for eligibility, confirmed eligible, included in the study, completing follow-up, and analysed	11	Line 214 and 220-222
		(b) Give reasons for non-participation at each stage	11	Line 223
		(c) Consider use of a flow diagram	Not applicable	Not applicable
Descriptive data	14*	(a) Give characteristics of study participants (eg demographic, clinical, social) and information on exposures and potential confounders	11	Line 214-215
		(b) Indicate number of participants with missing data for each variable of interest	Not applicable	Not applicable
		(c) *Cohort study*—Summarise follow-up time (eg, average and total amount)	Not applicable	Not applicable
Outcome data	15*	*Cohort study*—Report numbers of outcome events or summary measures over time	Not applicable	Not applicable
		Case-control study—Report numbers in each exposure category, or summary measures of exposure	Not applicable	Not applicable
		Cross-sectional study—Report numbers of outcome events or summary measures	11-13	Line 215-257
Main results	16	(*a*) Give unadjusted estimates and, if applicable, confounder-adjusted estimates and their precision (eg, 95% confidence interval). Make clear which confounders were adjusted for and why they were included	Not applicable	Not applicable
		(*b*) Report category boundaries when continuous variables were categorized	Not applicable	Not applicable
		(*c*) If relevant, consider translating estimates of relative risk into absolute risk for a meaningful time period	Not applicable	Not applicable

Other analyses	17	Report other analyses done—eg analyses of subgroups and interactions, and sensitivity analyses	11-13	Line 215-257
**Discussion**				
Key results	18	Summarise key results with reference to study objectives	13	Line 262-273
Limitations	19	Discuss limitations of the study, taking into account sources of potential bias or imprecision. Discuss both direction and magnitude of any potential bias	20	Line 419-423
Interpretation	20	Give a cautious overall interpretation of results considering objectives, limitations, multiplicity of analyses, results from similar studies, and other relevant evidence	14-20	Line 275-417
Generalisability	21	Discuss the generalisability (external validity) of the study results	20-21	Line 419-435
**Other information**				
Funding	22	Give the source of funding and the role of the funders for the present study and, if applicable, for the original study on which the present article is based	Reported in submission system	

*Give information separately for cases and controls in case-control studies and, if applicable, for exposed and unexposed groups in cohort and cross-sectional studies.

Note: An Explanation and Elaboration article discusses each checklist item and gives methodological background and published examples of transparent reporting. The STROBE checklist is best used in conjunction with this article (freely available on the Web sites of PLoS Medicine at http://www.plosmedicine.org/, Annals of Internal Medicine at http://www.annals.org/, and Epidemiology at http://www.epidem.com/). Information on the STROBE Initiative is available at www.strobe-statement.org.
