# Supplementary material for: Victory above all: the weight loss practices and perceptions of Chinese male kickboxers
Source: PeerJ. 2025 Jul 9;13:e19709. doi: 10.7717/peerj.19709 (PMC12255242; doi:10.7717/peerj.19709)
Supplement: Supplemental Information 4 [file peerj-13-19709-s004.pdf]

Codebook for Categorical Data in Raw Data

The codes representing all options are filled in the corresponding parentheses.

1. I have read the informed consent form above, and by completing this online survey, I agree to participate in this study.
(1) Yes
(2) No

3. Gender:
(1) male (2) female

4. Your sport
(1) Boxing
(2) Sanda
(3) Taekwondo
(4) Wrestling
(5) Judo
(6) Kickboxing
(7) Brazilian Jiu-Jitsu
(8) Ultimate Fighting Championship (UFC)
(9) Mixed Martial Arts (MMA)
(10) Sambo
(11) Muay Thai
(12) Other, please specify: _________

10. Please describe your achievements and participation in Boxing competitions to date.
School, Regional or city level competition (give some examples of regional or city level competitions)
（1） participated without winning medal （2） won a medal （3） never participated
State level (give some examples of state level competitions)
（1） participated without winning medal （2） won a medal （3） never participated
National level (give some examples of national level competitions
（1） participated without winning medal （2） won a medal （3） never participated
International level (give some examples of international level competitions
（1） participated without winning medal （2） won a medal （3） never participated

11. How many times did you compete in the last year (including non-official competitions)?
12. In how many competitions did you win medal in the last year (including non-official competitions)?

14. Did you change your weight class in the last two years?
(1) yes, in which weight classes did you compete?

(2) No, I compete in the same weight classes in the last two years.

15. Have you ever lost weight in order to compete? (Please do not select "No" just to avoid answering the remaining questions. If you do not wish to answer, please feel free to skip the questionnaire. We greatly appreciate your honest responses.)
(1) Yes. (Please continue answering the rest of the questionnaire)
(2) No, I have never cut weight to compete (thank you for your help-do not answer the following questions).

20. In how many days do you usually cut weight before competitions?
（1） 1-3 days
（2） 4-5 days
（3） 6-7 days
（4） 8-10 days
（5） 11-14 days
（6） 15-21 days
（7） other, __days (please specify)

22. In most cases, who guides your weight loss process? (Multiple choice question)
(1) Yourself (2) Sport coach (3) Strength and conditioning coach (4) Medical staff (5) Nutritionist (6) Parents (7) Other (please specify)

24. Using the scale below, please rate the amount of influence that each individual listed below has had on your weigth loss practices. (i.e.: who encouraged and taught you to lose weight) (check all items)
(1) Not influential
(2) little influential
(3) unsure
(4) some influential
(5) very influential

25. The table below presents several methods to lose weight rapidly. Using the table below, HOW OFTEN did you use each one of the following methods to lose weight before competitions? (Check all items).
(1) Always
(2) Sometimes
(3) Almost Never
(4) Never Used
(5) Do Not Use Any More

27. What are your reasons for engage in pre-competition weight loss? (Multiple choice question)
(1) To optimize athletic performance, such as increasing speed
(2) To compete against lighter opponents to increase the chances of winning

(3) My weight before the competition is higher than my normal weight, which is unfavorable for the competition
(4) Everyone else is cutting weight, so I have to do it too
(5) My coach told me to lose weight, so I have to do it
(6) Other (please specify)

28. How do you think weight loss affects your health?
(1) beneficial
(2) No impact
(3) detrimental

29. How do you think weight loss affects your performance in the competition?
(1) beneficial
(2) No impact
(3) detrimental

30. Do you think pre-competition weight loss leads to unfair competition?
(1) Yes, it leads to unfair competition
(2) No, the competition is still fair
(3) Not sure
