# Supplementary material for: Victory above all: the weight loss practices and perceptions of Chinese male kickboxers
Source: PeerJ. 2025 Jul 9;13:e19709. doi: 10.7717/peerj.19709 (PMC12255242; doi:10.7717/peerj.19709)
Supplement: Supplemental Information 5 [file peerj-13-19709-s005.pdf]

格斗运动员赛前减重调查

知情同意书

亲爱的运动员，

您好！

我们是上海体育学院 HP 研究团队，正在进行我国运动员赛前减重方式的调查。邀请您参加这个研究项目。目前，人们对运动员群体的赛前减重方式、幅度、动机、感受都知之甚少。这项研究将有助于科研人员和教练员对运动员的赛前体重规划有一个全面的了解，进而为运动员提供更科学的赛前减重方案。

调查可能需要花费您 15 分钟。全国所有参加省级及以上水平比赛的在役运动员均可填写。您不会因参与此项目而获得报酬。是否同意参与该项目完全取决于您。

参与这项研究可能带来的好处：更好地了解中国运动员的赛前减重方式、幅度、动机、感受，这将为训练科学研究人员和教练员提供证据基础，以便在未来的赛前训练中为运动员提供更科学更健康的减重方案。我们不能保证您将从这个项目中获得任何直接利益。

参与这项研究没有预期的风险。本次调查为匿名，所收集的资料只做研究用途，请您放心填答。请您根据自己的实际情况真实填写。

声明：

1. 您因参与该研究所发生的任何器质性损伤，上海体育大学不会为您支付医疗费用或提供其他经济补偿。您不会因签署本同意书而放弃任何法定权利。

2. 如果您对参与该研究有任何问题、困惑或投诉，或对您作为研究被试的权利持有任何疑问，您都可以与本同意书第一页所列的主要研究者和联系人联络。您也可以直接与上海体育学院科学研究伦理委员会联系，电话为 021-65508179，电子邮箱 lunli@sus.edu.cn

衷心感谢您的支持与配合！

黎涌明

上海体育学院 HP 研究团队

1、我已阅读上面的知情同意书，并通过完成此在线调查，我同意参与这项研究。

是　　　否

如果您未满 18 岁，请留下您的父母或监护人的联系方式，我们需要获得他们的知情同意。电话或邮箱：

____

请注意：由于这是一份匿名调查，因此您无需签名。如果您完成问卷，则同样视为您同意参与这项研究。

今天的日期：　　　年　　　月　　　日

一般信息

2、年龄：_________岁

3、性别：（ ）男，（ ）女

4、您当前的运动项目？（单选题）

（ ）拳击 （ ）散打 （ ）跆拳道 （ ）摔跤 （ ）柔道 （ ）自由搏击 （ ）巴西柔术 （ ）UFC（ ）综合格斗 MMA （ ）桑搏 （ ）泰拳 （ ）其它__________请说明

5、您几岁开始参加您当前项目的**训练**？ ______岁（填写年龄，勿写年份）

6、您几岁开始参加您当前项目的**比赛**？ ______岁（填写年龄，勿写年份）

7、您现在的体重是多少？__________公斤（kg）

8、您休赛季的体重是多少？__________公斤（kg）

9、您有多高？__________厘米。

运动成绩

10、请描述您至今为止在您当前项目的比赛中的成就和参与情况。（在每个比赛级别的对应格子打勾）（单选题）

来源	获得奖牌	参加但未获得奖牌	从未参加过
校级、区或市级比赛（如周口市比赛）			
省级比赛（如河南省比赛）			
国家级比赛（如全国比赛）			
国际级比赛（如世界杯、世锦赛、亚运会）			

11、过去十二个月里您参加过______次比赛？（包括非官方协会举办的正式比赛，不包括非正式的比赛，如队伍交流）

12、过去十二个月里，在上一题您参加的比赛中，您在______场比赛中获得了奖牌？

体重史和饮食模式

13、 您 2024 年参加哪个重量级别的比赛？__________公斤以下

14、 在过去的两年（2023 和 2024 年）里，您改变了您的参赛体重级别吗？（如去年打 52kg，今年打 60kg）

（ ） 是的，您参加了哪些重量级别的比赛？_______

（ ） 没有，我在过去两年中参加了相同的重量级比赛。

15、您是否曾经为了比赛而专门进行赛前减重？**（请勿为了不想回答剩余部分问卷而故意选择否，如您不想回答，请直接放弃填答。我们非常希望能够获得您的真实回答）**（单选题）

（ ）是的。（请继续回答问卷的其余部分）

（ ）不，我从来没有为了比赛而减肥（谢谢您的帮助，请不要再回答以下问题）。

个人赛前减重史（上一题选择“是”的继续填答）

16、你第一次为比赛减重是在几岁？_________岁。

17、至今为止，您为比赛而减去的 **最多体重的一次** 是多少公斤（kg）?__________公斤。

18、比赛前您 **通常** 减掉多少公斤（kg）?__________公斤。

19、在过去十二个月内您为了比赛减重了多少次_______次？

20、通常情况下，您的减重过程在比赛前什么时候开始？（单选题）

（_）1-3 天（_）4-5 天（_）6-7 天（_）8-10 天（_）11-14 天（_）15-21 天（_）其他情况__________天 (请说明)

21、将您减去的体重看作 100%，将减重的过程划分为三段：离称重还早，临近称重，称重前一天。您认为您通常在这三个时间段分别减去了总量（您一共减去的体重）100%的多少？（图片有助于进一步理解问题）

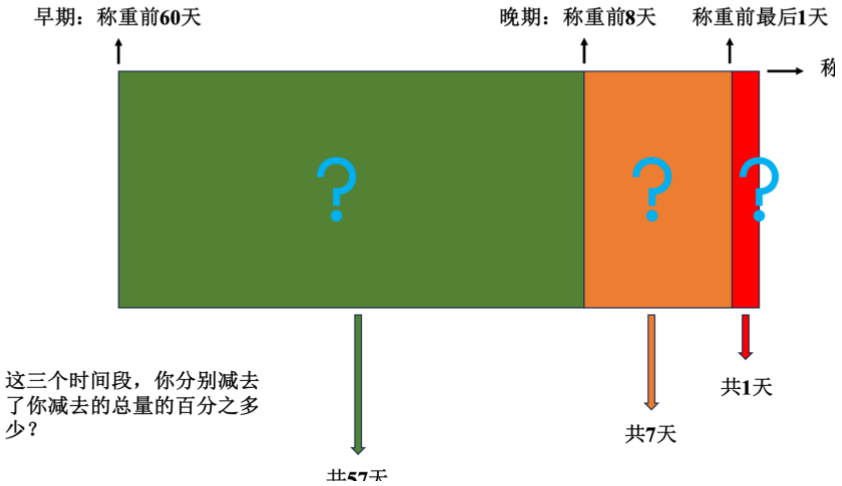

请注意：<u>**三部分加起来的总和必须为 100%**</u>。例如第一段（20%），第二段（35%），第三段（45%）

离称重还早阶段（称重前 60 天到称重前 9 天之间）减去的百分比为________%

临近称重阶段（称重前 8 天到称重前 2 天之间）减去的百分比为________%

赛前 1 天阶段（称重前 1 天到称重之间）减去的百分比为________%。　　请确保上面三个数字的总和是 100。

22、通常情况下，您的减重过程由谁指导？【多选题】

（ ）自己（ ）专项教练（ ）体能教练（ ）医务人员（ ）营养师（ ）父母（ ）其它____________（请说明)

23、通常情况下，称重过后，到比赛之前，您大约会恢复多少________kg 体重，然后再上去比赛？

24、请使用下面的量表，请对下面列出的每个人/事物对您的赛前**减重**实际影响程度进行选择。(即：鼓励和教您减重）（每一种来源勾选一种影响程度）。

来源	没有影响	影响不大	不确定	有一些影响力	非常有影响力
其他（不同项目的）运动员					
其他（同一项目的）运动员					
医生					
体能教练					
专项教练					
父母					
营养学家					
期刊文章					
书籍/杂志					
网络资源（如新闻、视频等）					
其他(请明)________________					

25、下表列出了几种快速减重的方法。阅读下表，选择您在比赛前使用以下不同方法减重的频率（每一种方法勾选一种频率，如禁食：经常；增加运动：从未使用）。

方法 \ 使用频率	经常使用	有时使用	很少，几乎从来没有用过	从来都没用过	以前用过，但已不再使用
逐步节食（2 周内或更长时间）					
每天不吃 1 或 2 餐					
禁食（整天不吃东西）					
限制液体（如水）摄入					
增加运动（比平时更多）					
有意地在加热的训练房进行训练					
蒸桑拿					
穿橡胶/塑料服（即控体重服）进行训练					
在不运动的情况下穿控体重服					
吐口水					
泻药					
利尿剂					
减肥药					
呕吐					
热水浴					

热盐水浴					
其他方法（请说明）：________________					

26、通常情况下，比赛结束后的第一周内，您大约会恢复多少体重？_________ 公斤/周。

六、对赛前减重的看法

27、您进行减重的原因？【多选题】

（ ）优化自身的运动表现，如速度更快

（ ）与低体重组的运动员竞争，提升获胜可能

（ ）比赛前体重高于我平常体重，不利于比赛

（ ）大家都减，我不得不减

（ ）教练让我减，我不得不减

（ ）其他，请说明_____________________________

28、您认为减重对于健康状况的影响是？（单选题）

（ ）提升健康水平

（ ）无影响

（ ）损害健康水平

29、您认为减重对于比赛表现的影响是？（单选题）

（ ）优化表现

（ ）无影响

（ ）降低表现

30、您认为赛前减重，是否导致比赛的不公平？（单选题）

（ ）是，导致比赛不公平

（ ）否，比赛仍然公平

（ ）不确定
