# Supplementary material for: Victory above all: the weight loss practices and perceptions of Chinese male kickboxers
Source: PeerJ. 2025 Jul 9;13:e19709. doi: 10.7717/peerj.19709 (PMC12255242; doi:10.7717/peerj.19709)
Supplement: Supplemental Information 6 [file peerj-13-19709-s006.pdf]

Questionnaire about pre-competition rapid weight loss

Informed Consent Form

Dear Athlete,

Hello!

We are the HP Research Team from Shanghai University of Sport, currently conducting a survey on the pre-competition weight loss methods of athletes in China. We invite you to participate in this research project. At present, very little is known about the weight loss methods, extent, motivations, and feelings of athletes before competitions. This research will help researchers and coaches gain a comprehensive understanding of pre-competition weight loss, thereby providing athletes with more scientifically-based weight loss strategies.

The survey will take approximately 15 minutes to complete. It is open to all active athletes who participate in provincial-level and higher competitions across the country. You will not receive any monetary compensation for participating in this project. Your participation in this project is entirely voluntary.

Potential benefits of participating in this study: By participating, you will contribute to a better understanding of pre-competition weight loss methods, extent, motivations, and feelings among Chinese athletes. This will provide a foundational reference for sports science researchers and coaches, helping them offer more scientifically sound and healthier weight loss plans for athletes in future training. We cannot guarantee any direct benefits from this project for you.

There are no expected risks associated with participating in this research. This survey is anonymous, and the data collected will only be used for research purposes. Please feel free to answer the questions truthfully based on your actual situation.

Statement:

1. Shanghai University of Sport will not cover any medical costs or provide financial compensation for any physical injury resulting from your participation in this research. By signing this consent form, you are not waiving any of your legal rights.
2. If you have any questions, concerns, or complaints about participating in this study or your rights as a research participant, you may contact the principal investigator and the contact person listed on the first page of this consent form. You may also directly contact the Ethics Committee for Scientific Research at Shanghai University of Sport at 021-65508179 or by email at lunli@sus.edu.cn.

We sincerely appreciate your support and cooperation!

Li Yongming

HP Research Team, Shanghai University of Sport

1. I have read the informed consent form above, and by completing this online survey, I agree to participate in this study.
( ) Yes ( )No

If you are under 18 years old, please provide your parent's or guardian's contact information, as we need to obtain their informed consent. Phone number or email:____________________

Please note: As this is an anonymous survey, you are not required to sign your name. Completing the questionnaire will be considered as your consent to participate in this study.

GENERAL INFORMATION. Today's Date:

2. Age: ________ years.

3. Gender: ( ) male ( ) female

4. Your sport
( ) Boxing ( ) Sanda ( ) Taekwondo ( ) Wrestling ( ) Judo ( ) Kickboxing ( ) Brazilian Jiu-Jitsu ( ) Ultimate Fighting Championship (UFC) ( ) Mixed Martial Arts (MMA) ( ) Sambo ( ) Muay Thai ( ) Other, please specify: __________

5. At what age did you begin to practice your current sport? ____years.

6. At what age did you begin to compete your current sport? ____years

7. How much do your current weight? ____kg

8. How much did you weigh in the last off-season (specify the year)? ____kg

9. How tall are you? ____m.

10. Please describe your achievements and participation in Boxing competitions to date.
School, Regional or city level competition (give some examples of regional or city level competitions)
( ) participated without winning medal ( ) won a medal ( ) never participated
State level (give some examples of state level competitions)
( ) participated without winning medal ( ) won a medal ( ) never participated
National level (give some examples of national level competitions
( ) participated without winning medal ( ) won a medal ( ) never participated
International level (give some examples of international level competitions
( ) participated without winning medal ( ) won a medal ( ) never participated

11. How many times did you compete in the last year (including non-official competitions)?___
12. In how many competitions did you win medal in the last year (including non-official competitions)?

WEIGHT HISTORY AND DIET PATTERNS.

13. In which weight class do you compete? Under____kg

14. Did you change your weight class in the last two years?
( ) yes, in which weight classes did you compete?_______
( ) No, I compete in the same weight classes in the last two years.

15. Have you ever lost weight in order to compete? *(Please do not select "No" just to avoid answering the remaining questions. If you do not wish to answer, please feel free to skip the questionnaire. We greatly appreciate your honest responses.)*
Yes. (Please continue answering the rest of the questionnaire)
No, l have never cut weight to compete (thank you for your help-do not answer the following questions).

Personal Pre-Competition Weight Loss History (Please continue filling out if you selected "Yes" in the previous question)

16. At what age did you begin to cut weight for competitions? ______years old.

17. What is the **MOST WEIGHT** that you have cut to compete in your career?______kg.

18. How much weight do you **usually** cut before competitions?______kg.

19. How many times did you cut weight to compete last season (specify the year)? ______

20. In how many days do you **usually** cut weight before competitions?
（_） 1-3 days
（_） 4-5 days
（_） 6-7 days
（_） 8-10 days
（_） 11-14 days
（_） 15-21 days
（_） other, __days (please specify)

21. Please divide the weight loss process into three stages: early phase, late phase, and final phase. Consider the total amount of weight you lost as 100%. How much of the total weight loss (100%) do you usually lose in each of these three stages? (Images may help further clarify the question)

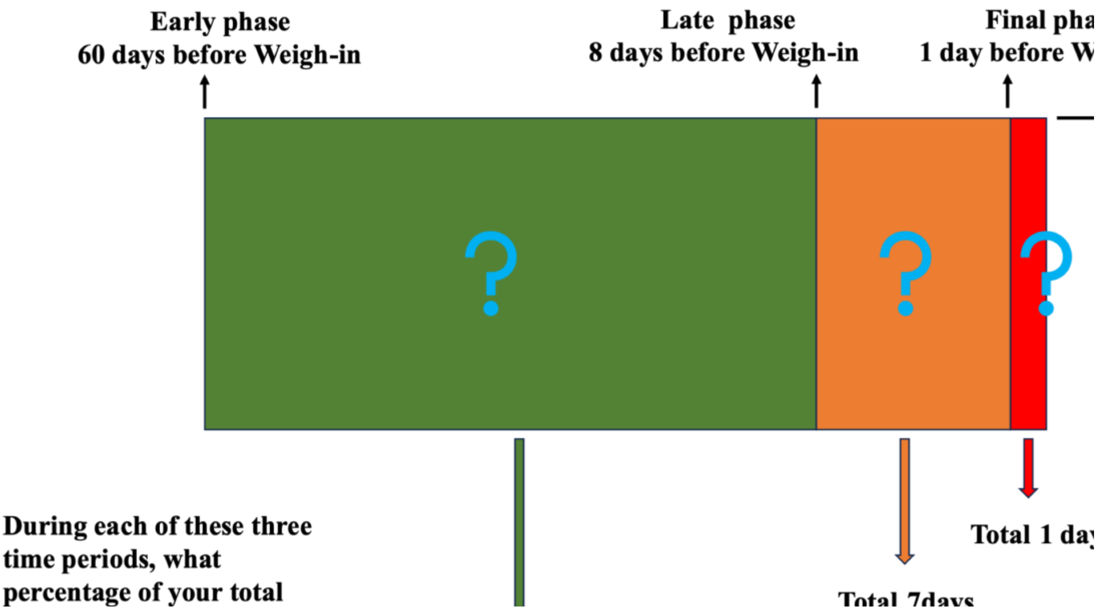

During the early phase of weight loss (from 60 days to 9 days before the weigh-in), the percentage of total weight lost is ________%.

During the late phase (from 8 days to 2 days before the weigh-in), the percentage of total weight lost is ________%.

During the final day phase (from 1 day before the weigh-in to the day of weigh-in), the percentage of total weight lost is ________%.

Please ensure that the total of these three percentages adds up to 100%.

22. In most cases, who guides your weight loss process? (Multiple choice question)

( ) Yourself ( ) Sport coach ( ) Strength and conditioning coach ( ) Medical staff ( ) Nutritionist ( ) Parents ( ) Other (please specify)

23. Usually, after weighing in, how much weight do you typically recover before the competition and then compete?

24. Using the scale below, please rate the amount of influence that each individual listed below has had on your weigth loss practices. (i.e.: who encouraged and taught you to lose weight) (check all items)

1 2 3 4 5

Not influential; little influential; unsure; some influential; very influential

source	Influence
Other athlete (different sport)	
Other athlete (same sport) /training colleague	
Physician/doctor	
Strength and conditioning coach /physical trainer	
Sports coach	
Parents	
Dietitian/ nutritionist	
Journal articles	

Book/ magazines	
Internet sources	
Other. Explain________________	

25. The table below presents several methods to lose weight rapidly. Using the table below, HOW OFTEN did you use each one of the following methods to lose weight before competitions? (Check all items).

methods \ frequency	Always	Sometimes	Almost Never	Never Used	Do Not Use Any More
Gradual dieting					
Skipping meals					
Fasting					
Restricting fluid ingestion					
Increased exercise					
Training in a heated room					
Sauna					
Training in plastic suits					
Use plastic suit all-day					
Spitting					
Laxatives					
Diuretics					
Diet pills					
Vomiting					
Hot water immersion					
Hot saltwater immersion					
Others					

26. How much weight do you **usually** regain in the week following a competition?__kg/week.

27. What are your reasons for engage in pre-competition weight loss? (Multiple choice question)
( ) To optimize athletic performance, such as increasing speed
( ) To compete against lighter opponents to increase the chances of winning
( ) My weight before the competition is higher than my normal weight, which is unfavorable for the competition
( ) Everyone else is cutting weight, so I have to do it too
( ) My coach told me to lose weight, so I have to do it
( ) Other (please specify)

28. How do you think weight loss affects your health?
( ) beneficial
( ) No impact
( ) detrimental

29. How do you think weight loss affects your performance in the competition?

( ) beneficial

( ) No impact

( ) detrimental

30. Do you think pre-competition weight loss leads to unfair competition?

( ) Yes, it leads to unfair competition

( ) No, the competition is still fair

( ) Not sure
